# Implementation of an Intraoperative Augmented Reality Environment for Custom-Made Partial Pelvis Replacements—A Proof of Concept and Initial Results

**DOI:** 10.3390/jpm16020124

**Published:** 2026-02-21

**Authors:** Yannik Hanusrichter, Carsten Gebert, Sven Frieler, Marcel Dudda, Arne Streitbuerger, Jendrik Hardes, Lee Jeys, Martin Wessling

**Affiliations:** 1 Department of Tumour Orthopaedics and Revision Arthroplasty, Orthopaedic Hospital, Volmarstein, Lothar-Gau-Str. 11, 58300 Wetter, Germany; 2 Center for Musculoskeletal Surgery, University Hospital of Essen, 45147 Essen, Germany; 3 Department of Orthopedics and Tumor Orthopedics, Muenster University Hospital, 48149 Muenster, Germany; 4 Department of Trauma and Orthopedic Surgery, BG University Hospital Bergmannsheil, Ruhr-University Bochum (RUB), 44789 Bochum, Germany; 5 Department of Trauma Surgery, University Hospital Essen, 45147 Essen, Germany; 6 Department of Orthopedic Oncology, University Hospital Essen, 45147 Essen, Germany; 7 Department of Oncology, The Royal Orthopaedic Hospital, Birmingham B31 2AP, UK; 8 Faculty of Health Sciences, Aston University, Birmingham B4 7ET, UK

**Keywords:** augmented reality, individual implants, partial pelvis replacement, revision hip arthroplasty, rTHA, navigation, custom-made implants

## Abstract

**Background**: The use of augmented reality (AR) in orthopaedics is growing rapidly but is mainly limited to pre-operative planning and teaching. This study is one of the first to describe the intraoperative application within revision arthroplasty for the positioning of customised partial pelvic replacements. **Methods**: In a proof-of-concept study an AR environment was used during surgery in 11 cases to enhance implant positioning. Postoperatively, a voxel-based CT deviation analysis was carried out to determine the COR deviation and the cup plane deviation angle. Additionally, digital implant superimposition was conducted. **Results**: Implantation was possible in all cases with a mean COR deviation vector of 4.2 (SD 2.5; 1.2–9.3) mm and a cup plane deviation angle of 4.4 (SD 2.5; 0.7–8.1)°. The implant analysis showed a superimposition of 0.69 (SD 0.15; 0.38–0.88) (Dice-Score calculation). **Conclusions**: This study is able to report promising results for AR in orthopaedic surgery, showing improved intraoperative feedback in complex operations, resulting in increased accuracy. However, the integration of AR poses a new challenge to the surgical team, especially because the AR users are facing a significantly increased level of intraoperative stress. Further development of this auspicious tool, as well as a conceivable combination with navigation, is necessary to facilitate broader usage.

## 1. Introduction

Custom-made partial pelvic replacements (PPR) have shown to be a valuable solution in severe acetabular defects, achieving adequate functional outcome as well as anatomical reconstruction [1,2]. As this technique is now being incorporated into the treatment algorithm for revision total hip arthroplasties (rTHA), the evidence base is becoming sound. Several studies, including meta-reviews and medium-term results, have now been published [3]. Although treatment algorithms and designs still differ according to fixation philosophy, it has been shown that with meticulous planning, a high degree of positioning accuracy can be achieved [4]. However, there are still margins of up to 1 cm for centre of rotation (COR) deviation (planned vs. achieved) as well as up to 5–10° in angulation present [5,6]. These errors may lead to deviation in the intramedullary location and screw tip position of the main fixation, as longer (>7 cm) implants (e.g., screws or stems) are usually used here. For example, a deviation of 5° for an 8 × 80 mm screw will result in a deviation of 8 mm for the screw tip, resulting in a possible extra-osseous position with associated risks such as neural or vascular damage. Various tools have been developed to increase the accuracy of implant and fixation positioning; life-sized models, as well as patient-specific instruments (PSI) and instructions, provide valuable intraoperative support [7,8]. However, best practice algorithms are still to be established. Implementation of new technologies might increase the safety and accuracy of these highly complex procedures and, ideally, improve the patient’s functional outcome. Augmented reality applications have been proposed for primary hip arthroplasties to enable a detailed inclination and anteversion adjustment [9]. Additionally, some case reports have been published, transferring these concepts to revision arthroplasty settings, enabling resection planning as well as positioning of custom-made implants [10,11]. To facilitate a broader application, we present a proof-of-concept study, implementing a therapy protocol for the use of an intraoperative augmented reality (AR) environment for custom-made partial pelvic replacements using different models, as well as CT scan feedback to facilitate a detailed visualization. Additionally, a detailed postoperative 3D analysis was conducted to determine the implant positioning accuracy.

## 2. Materials and Methods

The study was conducted on eleven consecutive PPR surgeries between November 2023 and July 2024, performed by three senior surgeons in a referral centre. The AR environment was used by the same surgeon in all cases (YH), who, as the AR user, was assisting the operation, supporting the first surgeon. The AR environment was used as a comparison layer, not as a direct navigation in the intraoperative situs. The exact use cases are shown in the Supplementary Video S1. The implants and virtual models (.stl data for AR) were provided by the PPR manufacturer (Implantcast, Buxtehude, Germany). The inclusion criterion was the implantation of a PPR as a revision arthroplasty. The indication consisted of aseptic loosening in six cases and periprosthetic joint infection (PJI) in five cases. In order to make a more substantial statement, a comparison was made with a collective already published by our work group (*n* = 45; aseptic loosening *n* = 23, PJI = 20, fracture *n* = 2), which was analysed in a similar manner [5]. Base parameters for both collectives are shown in Table 1. The design philosophy in both groups was similar, using a monoflange design, which was already published in detail [5]. Ethical approval was obtained prior to the investigation from the local ethics committee (reference number 21-10438-KOBO). Additionally, individual-informed consent was obtained for the intraoperative AR usage.

## 3. Study Design

Based on the preoperative CT scan, a segmentation of the bone, as well as needed preparation for the PPR, was conducted by an engineer. In close cooperation with the surgeons, an individual implant was planned. The implant design philosophy, as well as the CT scan protocol, was based on an already published design algorithm using CAD-CAM engineering [5]. After the implant design was completed, several files were uploaded in the augmented reality environment (VSI Holomedicine^®^, ApoQlar medical, Hamburg, Germany) on a HoloLens 2 (Microsoft, Redmond, WA, USA). This consisted of data already used for the engineering process: (I) .stl data of the pelvis with the needed preparation for implant fitting; (II) merged .stl of the pelvis, the implant as well as the screw positioning; and (III) merged .stl data of the pelvis, the implant and extrapolated k-wires for the main fixation. Additionally, a 3D model of the CT scan and fixed CT views with a schematic drawing of the implant could be provided. Surgical procedure as well as implant properties were in line with an already published algorithm for PPR; therefore, only the main aspect will be mentioned [5,12]: (I) Reconstruction focused on centre of rotation (COR), acetabular inclination (IC) and anteversion (AV); (II) in aseptic cases a two-stage exchange was conducted if extensive metal artifacts prohibited a one-stage planning; (III) main fixation was achieved by cranial fixation with 8 mm intramedullary screws and/or highly porous 9 mm stem; (IV) approved design was manufactured using additive layer manufacturing with highly porous titanium metal (Epore^®^, Implantcast, Germany) to achieve bone ingrowth and biological fixation; and (V) after fixation of the PPR implantation of a cemented dual-mobility cup (2M, Implantcast, Germany) was performed, allowing fine tuning of inclination and anteversion.

**Figure 1:**
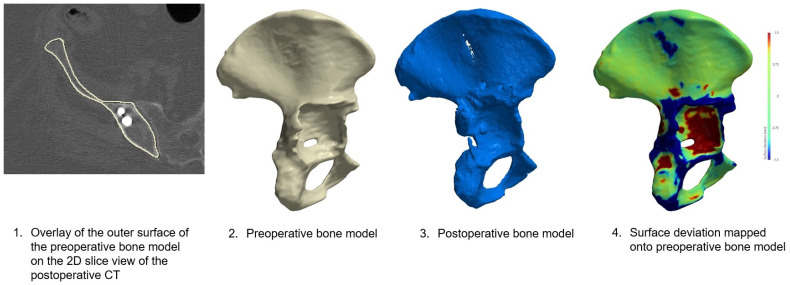
Greyscale matching algorithm for the pre- and postoperative CT scans, resulting in a matched bone for implant analysis; Image **1**: Manual verification conducted on 2D slices between the pre- and postoperative scans; Image **2**: Preoperative segmented bone model; Image **3**: Postoperative segmented bone model; Image **4**: Surface deviation analysis of the fused images.

Postoperatively, CT scans as well as X-rays were conducted to assess implant and fixation accuracy. The X-rays were measured for contralateral COR deviation using a mirrored COR and a 2D vector. In addition, a postoperative workflow was established to facilitate the best possible matching of the pre- and postoperative CT scans for further analysis (Simpleware software, Synopsys Inc., Sunnyvale, CA, USA): (I) Segmentation of the post-op CT utilizing automated segmentation tools powered by Machine Learning (ML) algorithms (Simpleware-AS Ortho) followed by manual refining and correcting; (II) Image registration with first shrink-wrapping the planning CT and post-op CT to the affected pelvic bone and using a grayscale-matching algorithm to superimpose both scans; and (III) Visual inspection to check for alignment with an overlay of the virtual bone models conducting a surface deviation analysis mapped onto the preoperative bone model as well as using 2D slices (Figure 1).

**Figure 2:**
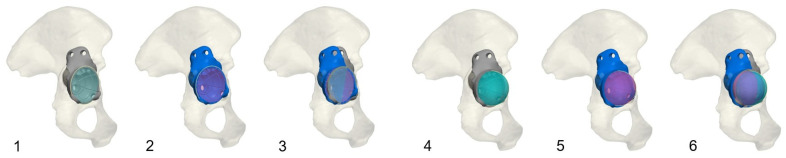
Cup plane as well as COR deviation analysis after merged mapping of the preoperatively planned and postoperatively achieved implant position; Image **1**–**3**: Cup angle deviation analysis, showing the pre- and postoperative and fused image; Image **4**–**6**: Same analysis conducted for the COR deviation.

Then, a deviation analysis between the planned and postoperative achieved PPR position, as well as COR distance deviation (measured as a 3D Vector) was conducted. Additionally, the cup plane was registered, and an angulation deviation analysis was conducted, resulting in a degree measurement representing the three-dimensional tilt of the cup plane (Figure 2).

**Figure 3:**
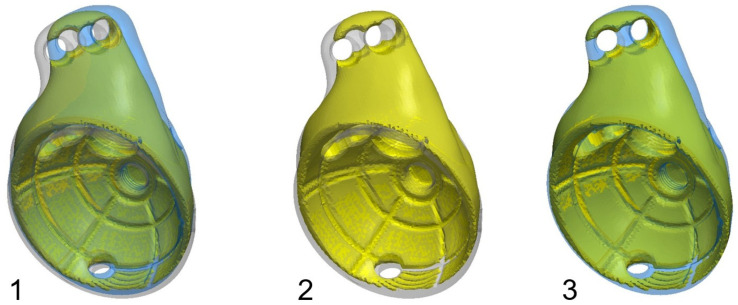
Overlapping voxel analysis for dice score calculation; Image **1**: preoperative (grey) and postoperative (blue) PPR positioning, showing the overlapping voxels (green); Image **2**: Difference between the preoperative (grey) and overlapping voxel (yellow); Image **3**: Difference between the preoperative (blue) and overlapping voxels (green).

To further analyse the accuracy of the implant positioning, a Dice-similarity coefficient (DSC) was calculated by assigning the same number of voxels in each implant based on the following calculation and a superimposition of the planned to achieved position: DSC = 2 × |A ∩ B|/(|A| + |B|). A score of 1 indicated perfect alignment between the planned and implanted mask; a score of 0 indicated no overlap at all. The superimposing images are shown in Figure 3.

## 4. Statistical Analysis

Data analysis was performed using the Statistical Package for Social Sciences Software (IBM SPSS Statistics Version 24, Chicago, IL, USA). Descriptive statistical results were recorded to describe comorbidities, complications, and previous procedures. The Shapiro–Wilk test was performed to determine non-normal/normal distribution. T-test was used for parametric, Mann–Whitney U for non-parametric values in univariate analysis.

## 5. Results

**Figure 4:**
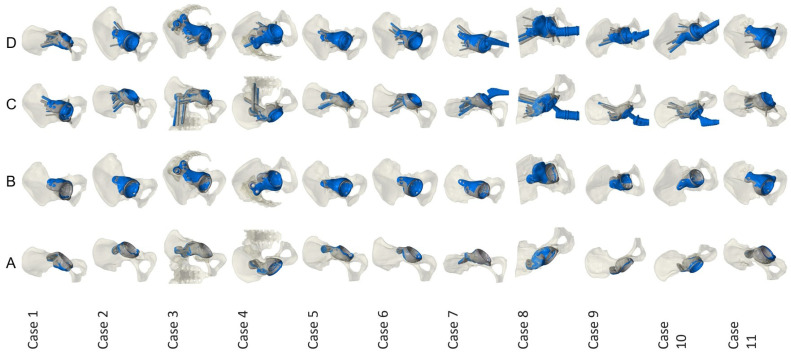
3D analysis for all cases showing preoperatively planned (grey) and postoperatively achieved (blue) implant position; Row A/B: anterior and lateral view of the implant; Row C/D: anterior and lateral view of the implant with fixation analysis.

Implantation was carried out in all cases with a mean operation time of 221 (SD 70; 143–370) minutes as a two-stage approach in 10 cases and a one-stage approach in one case; there was no significant difference from the previously published collective (*p* = 0.077). All operations were carried out by three senior surgeons (CG, MW, YH). Stem retention was possible in all aseptic cases; in septic cases, a complete exchange was conducted. Acetabular defect assessment showed three Paprosky IIA and eight Paprosky IIIB defects with three pelvic discontinuities. Additional iliosacral fixation was carried out in two cases, using the AR orientation as well as intraoperative fluoroscopy. Postoperative X-ray analysis showed a mean COR deviation to the contralateral side of 4.5 (SD 2.1; 2–8) mm. The 3D mapping analysis resulted in a mean COR deviation planned vs. achieved of 4.2 (SD 2.5; 1.2–9.3) mm, compared to the published collective with a deviation of 15.5 (SD 8.5; 4–32) mm (*p* < 0.001; Mann–Whitney-U). (5) Cup angle plane analysis showed a deviation of 4.4 (SD 2.5; 0.7–8.1)°. Dice score calculation resulted in a mean value of 0.69 (SD 0.15; 0.38–0.88). Implant superimposition for each case is shown in Figure 4.

Mean intraoperative AR setup time was 12 (SD 9; 5–30) minutes with a significant reduction during the study period. There were no extracortical screw/stem misplacements and no cortical breaches. The main fixation screw length was implanted as planned in all AR cases; in contrast, a shorter screw length had to be selected in 8 (17%) cases in the comparison group. While not the focus of the study, we are able to report a follow-up of 18 (SD 4; 12–26) months, and all implants survived. The functional outcome, analysed using the Harris Hip Score, showed a mean of 72 (SD 14; 45–91) points. During the follow-up, one patient received a constrained liner due to instability, and one patient underwent a Debridement, antibiotics, and implant retention procedure due to a postoperative periprosthetic joint infection. Detailed parameters for the collective, as well as a comparison, are shown in Table 2. Intraoperative usage is shown in the Supplementary Video S1.

## 6. Personal Experience and Workflow

As there are only sparse publications on the actual usage of augmented reality within orthopaedic surgeries, the authors would like to present their personal experience and obstacles during the implementation, as several factors have to be considered [13,14,15]. The learning curve is steep and requires meticulous pre-operative training of a tech-savvy person due to the early stage, as salvage solutions are often required based on technical limitations. Additionally, transferring the three-dimensional preoperative planning into the operating theatre as a visualisation in a real surgery is demanding. For example, preoperative fusing of the images has to be conducted, requiring additional time. While software applications exist (e.g., Spectomed Specto Medical, Basel, Switzerland), a case-by-case solution is needed. As the application is solely based on a single-person experience, we are not able to conduct any objective analysis of the learning curve. To give more insights, the preoperative training before the first cases was three hours, with an intraoperative setup time of 30 min. Additionally, first assistance was covered by a third surgeon for the first two cases, and afterwards, no extra staff was required. In the last case, an on-the-fly setup with no downtime and a full first assistance of the AR user was possible. As with each new technique, AR setup puts an increased strain not only on the user but on the whole operation team, as additional hardware is needed (HMD, extra monitor, or live streaming). If the HMD is used in the later stage of the operation, it has to be put on by an assistant, requiring extra steps and instructions; however, to reduce the stress on the AR user, we would recommend this technique. Following these steps, we were able to decrease intraoperative set-up time dramatically, as seen in the results from 30 to 5 min. During implementation, although a change in the intraoperative process was conducted and additional devices were used, we did not encounter any specific situations requiring more focus to ensure sterility.

In our implementation setup we mainly used the AR environment for five parameters: (I) As a comparison between the AR model and the intraoperative tactile situation gave surprisingly good feedback, detailed preparation was possible with a “on the fly” comparison (II) Due to the extrapolated k-wire display, a comparison of the intraoperative angle and the model was possible (III) Analogous to the preparation, a comparison of the flange was possible after implant positioning (IV) Screw placement and angle was also comparable using the same methods, allowing for a precise placement (V) Live streaming on an extra monitor was conducted, ensuring a discussion between the AR user and the head surgeon, enabling detailed visible feedback. It is worth mentioning that the usage was mainly based on a comparison, not a direct navigation in the intraoperative field.

As stated, technical limitations must be considered. First, there is no registration possible, and the inaccuracy based on “manual” matching is too high to facilitate a superimposition on the patient; therefore, we positioned the AR models “flying” above the operation table, pulling them into the field of view as needed. The second drawback is a darkened field of view due to the HMD, requiring explicit intraoperative light adjustment; additionally, head-mounted light sources might be viable. This aspect further reduces the usefulness of the superimposition of the models at the site of the operation, as the intraoperative light makes the AR models more translucent, impairing a mapped comparison. Another aspect is the provided software environment, which registers the hand gestures conducted during the operation, sometimes resulting in unwanted instructions for the HMD. Therefore, we recommend using the HMD as a varifocal, changing between the AR environment and the operating site by adjusting the field of view (above and on the patient). Close communication between the first and second surgeon is necessary to achieve appropriate feedback, especially as the models are not visible to the first surgeon, or only as a streaming in 2D; this has to be trained in detail. This is one of the main positive aspects of using a HMD in our experience, as slight differences could better be compared in models, which can be manipulated by the surgeon instead of a 2D monitor which is guided by an extra assistant. Additionally, by bringing the information directly to the table as a “second” layer, the feedback was improved, compared to sterile life-sized models. There were no errors in intraoperative use during this study, but as this was a first implementation, two experienced surgeons performed each operation, providing a ‘fall back’ to conventional methods in the event of a system error.

**Figure 5:**
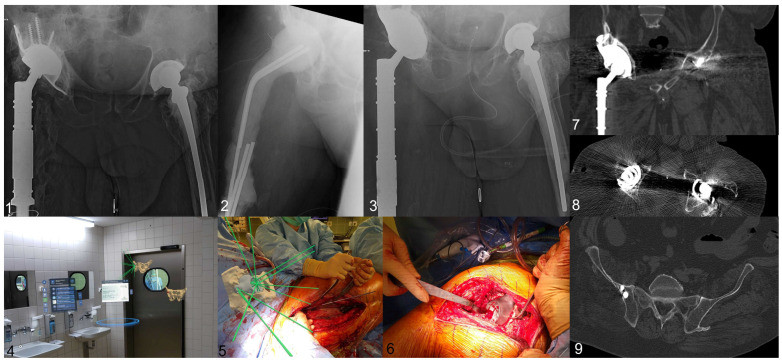
Exemplary case (no. 8) Image **1**: preoperative image showing an extensive paprosky IIIb defect, simultaneously a PJI was present; Image **2**: X-ray after explantation, a proximal femur spacer was implanted; Image **3**: postoperative Image; Image **4**: preoperative setup of the AR environment; Image **5**: comparison between planning and intraoperative positioning; Image **6**: final implant position; Image **7**–**9** postoperative CT scan.

Due to the additional layers and the required feedback from the AR user, the intraoperative stress level is significantly increased, making a complex surgery even more demanding, especially if the environment is used as mentioned above, requiring a “double layer” awareness of the AR user. This factor should not be underestimated; therefore, the use of the “main” surgeon is, at the moment, not feasible in our experience, as too many factors must be assessed at the same time. An exemplary case is shown in Figure 5. Supplementary Video S1 Material is included to demonstrate the intraoperative usage in several steps.

## 7. Discussion

In this study, we are able to report one of the first use cases of an intraoperative augmented reality environment for revision arthroplasty and custom-made implants on a head-mounted device (HMD). Custom-made implants for revision arthroplasty remain a demanding surgery in terms of indication, preoperative planning, and intraoperative execution [3].

As other studies and our own work have shown, PPR can be implanted with a high accuracy, however COR deviation analysis conducted in the studies has shown a deviation of 1–2 cm as a mean value if a 3D vector was calculated, mean deviation for each axis (x, y and z) differs between 4 and 8 mm in the published studies [4,5,16]. As known from biomechanical studies, COR reconstruction is the main factor for a sufficient postoperative function; therefore, even small deviations that have to be corrected by head length correction result in a sub-optimal force vector [17,18]. Therefore, the use of AR appears to further enhance intraoperative accuracy, making the detailed preoperative three-dimensional planning available at the operating table.

The use of AR within the orthopaedic spectrum has emerged in recent years, particularly in spinal and neurosurgery [19]. However, most applications are limited to preoperative planning, modelling studies, or teaching [15]. Focusing on joint arthroplasty, some studies have investigated the AR-supported cup placement for primary arthroplasties. Sun et al. conducted a meta-review for hip surgery, showing use cases in 30 studies, which mainly included preoperative simulation and training or cup placement for primary arthroplasties [20]. In the same manner, Liu et al. published a surgical workflow for hip resurfacing using augmented reality-based navigation on a sawbone model, showing a comparable result to navigation systems [21]. Ogawa et al. analysed cup placement with an augmented reality system, superimposing the optimal placement through a smartphone display, showing an increased accuracy for anteversion (*p* < 0.0001) [22]. In a follow-up study, the group published the results of 46 patients as a randomized controlled trial for either AR or conventional cup placement, showing a significant increase in accuracy for cup placement, albeit the differences of 1.5° and the clinical impact are debatable [23].

In a recent meta-review by Lex et al., further investigation was conducted for AR in orthopaedic surgery, stating an anticipated increase in the next few years; however, the review showed that, although all the studies carried out were able to achieve a potential improvement, this was only obtainable through a substantially increased workload, particularly in the preoperative preparation phase [13]. Therefore, the increased surgery and preoperative set-up time do not currently support its regular use in primary arthroplasties, especially as excellent results can already be achieved with conventional methods. The often-mentioned reduced X-ray exposure is also neglectable for primary arthroplasties. Opposing this due to the high effort involved and the possible complication rate for PPR, as well as the still lower accuracy compared to primary operations, further improvement of the procedure is desirable, especially considering the time already invested in each case [2]. As the intraoperative AR environment used in this study accesses the data required for production, the additional time required seems reasonable, especially as all information is available to the surgical team during the procedure. In the present study, the augmented reality environment was used as an additional intraoperative reference rather than a replacement for conventional imaging. Consequently, a comparable number of intraoperative fluoroscopic images was obtained, and no reduction in radiation exposure was observed.

As already mentioned above, AR usage results in an increased intraoperative burden for the user; however, with increased usage as well as reduced intraoperative time, we are able to report acceptable subjective stress levels similar to other conducted studies [24]. However, as already stated, we are not able to provide any measurable data for this parameter and base it only on individual impressions during the study period. Still, we recommend AR usage only for the second surgeon at the current state.

To date, the use of AR is still limited and at an early stage. Marker-less navigation is not feasible due to the small margin for errors, which cannot be provided by the HMD software at the moment [15]. Especially since the intraoperative movement of the patient cannot yet be mapped by an isolated augmented reality environment [14]. Therefore, navigation is not possible without other hardware, making a patient and instrument navigation marker necessary, resulting in a complex combined AR-navigation setup [13]. Intraoperative overlay of the augmented reality models is correspondingly more difficult, as the described inaccuracy is transferred, and the reduced visibility also has an impact, as stated above.

It is worth mentioning that AR environments for primary arthroplasties have been established, transferring the information on robotic systems to the surgeon by using HMD, e.g., ARVIS^®^ (Enovis, DE, USA). However, these systems suffer from the same problems encountered in robotics used for revision arthroplasty and especially PPR, as a “standard” approach is not feasible on surgeries treated on a case-by-case basis.

Future applications for the use of AR in revision arthroplasty and complex orthopaedic surgery include the ease of recording high-quality videos of surgery for teaching, training and documentation, and also open up the possibility of virtual remote surgeon-to-surgeon collaborations for difficult cases [13].

Several limitations have to be mentioned for this study. As it was conducted as a proof of concept, the cohort is small and consists of a single-centre experience. We also want to emphasize again that the AR environment was used as a secondary guidance, with no real navigation markers or CT-based intraoperative matching conducted. Additionally, as the main focus was intraoperative assessment and accuracy, we are only able to report short-term follow-up with limited functional result analysis. As the usage of AR is on the rise, we recommend more multi-centre and multi-user studies to further facilitate our findings. However, as this study demonstrates a successful intraoperative application, it provides a basis for further research.

## 8. Conclusions

The use of augmented reality is still in its infancy, particularly in orthopaedics. However, there are signs of rapid development that offer the potential for this technology to become a valuable tool for highly complex surgeries. In our study, we are able to provide (I) a first-time application for revision arthroplasty and (II) a voxel-based pre- to postoperative matching, allowing for a detailed implant accuracy analysis with promising results.

Historically, in orthopaedic surgery, intraoperative X-ray has brought the ability to directly control surgical outcomes into the operating theatre. The next step, with the use of navigation systems and three-dimensional planning, enables the transfer of preoperative planning to the surgical procedure. In the future, augmented reality may bring the possibility of combining these technologies and putting them all in the surgeon’s hands.

## Figures and Tables

**Table 1:** Patient parameters.

Parameter	Conventional Group	AR Group	*p*-Value
*n*	45	11	
Female (%)	38 (84)	8 (73)	0.367
Mean Age (SD; range) (years)	70 (13; 30–88)	70 (10; 58–85)	0.642
Mean BMI (SD; range)	27 (6; 18–42)	30 (6; 20–38)	0.205
Mean ASA Score (SD; range)	3 (0.5; 2–4)	3 (0.5; 2–3)	0.763
Mean surgery time (SD; range)	206 (64; 95–387)	221 (70; 143–370)	0.773
Mean additional 6,5 mm screws (SD; range)	4 ± 1.7 (2–9)	3 (1.2; 0–4)	0.297
Mean 6.5 mm screw length (SD; range) (mm)	40 (12; 20–90)	31 (10; 20–60)	0.001

**Table 2:** Patient analysis.

Patient No.	Age	Indication	Paprosky Classification	Primary Fixation	Used/Planned Length (mm)	2D Contralateral COR Deviation (mm)	3D COR Distance (mm) Planned–Achieved	Cup plane Angle (°) Planned-Achieved	Dice Score
1	72	aseptic loosening	3A	8.0 screw	70/60	6	9.3	1.1	0.38
				9.0 mod. stem	50/50				
2	85	aseptic loosening	3B ^2^	8.0 screw	60/60	7	4.1	3.8	0.67
				8.0 screw	60/60				
3	72 ^1^	aseptic loosening	3B ^2^	8.0 screw	80/80	5	8.1	1.6	0.53
				8.0 screw	70/70				
				9.0 mod. stem	100/100				
				9.0 mod. stem	60/60				
4	78 ^1^	periprosthetic joint infection	3B ^2^	8.0 screw	70/70	7	2.2	4.1	0.76
				8.0 screw	70/70				
				9.0 mod. stem	90/90				
				9.0 mod. stem	60/60				
5	82	aseptic loosening	3B	8.0 screw	70/70	5	5.6	8.1	0.68
				9.0 mod. stem	70/60				
6	68	periprosthetic joint infection	3B	8.0 screw	70/60	2	2.2	4.8	0.78
7	59	periprosthetic joint infection	3B	8.0 screw	80/80	2	2.1	0.7	0.87
				9.0 mod. stem	80/80				
8	58 ^3^	aseptic loosening	3A	8.0 screw	80/80	/	2.8	7.7	0.81
				9.0 mod. stem	80/80				
9	58	aseptic loosening	3B	8.0 screw	60/60	3	4.2	4.8	0.5
				9.0 mod. stem	80/70				
10	63	periprosthetic joint infection	3B	8.0 screw	70/70	3	1.2	5.7	0.88
				9.0 mod. stem	90/90				
11	77	periprosthetic joint infection	3A	8.0 screw	60/60	6	4.9	5.9	0.7
				9.0 mod. stem	50/50				
					Mean (SD; range)	4 (2; 2–7)	4.4 (2.5; 0.7–8.1)	4.4 (2.5; 0.7–8.1)	0.69 (0.15; 0.38–0.88)

^1^ Iliosacral-fixation ^2^ Pelvic discontinuity ^3^ native COR not planned due to dysplasia.

## Data Availability

The datasets used and/or analysed during the current study are available from the corresponding author on reasonable request.

## Supplementary Materials

The following supporting information can be downloaded at: https://www.mdpi.com/article/10.3390/jpm16020124/s1, Video S1: Intraoperative augmented reality use examples.
